# Comparison of HIV drug resistance profiles across HIV-1 subtypes A and D for patients receiving a tenofovir-based and zidovudine-based first line regimens in Uganda

**DOI:** 10.1186/s12981-020-0258-7

**Published:** 2020-01-31

**Authors:** Alisen Ayitewala, Fred Kyeyune, Pamela Ainembabazi, Eva Nabulime, Charles Drago Kato, Immaculate Nankya

**Affiliations:** 1 Center for AIDS Research Laboratories, Joint Clinical Research Center, P.O. Box 10005, Kampala, Uganda; 2 School of Biosecurity, Biotechnology and Laboratory Sciences, College of Veterinary Medicine Animal Resources and Biosecurity, Makerere University, P. O. Box 7062, Kampala, Uganda

**Keywords:** HIV drug resistance, HIV-1 subtype, ART drug regimen

## Abstract

**Background:**

Resistance to antiretroviral drugs is a major challenge among Human Immunodeficiency Virus (HIV) positive patients receiving antiretroviral therapy (ART). Mutations that arise as a result of this are diverse across the various drugs, drug classes, drug regimens and subtypes. In Uganda, there is a paucity of information on how these mutations differ among the different drug regimens and the predominant HIV-1 subtypes. The purpose of this study was to determine mutation profile differences between first-line drug regimens: TDF/3TC/EFV and AZT/3TC/EFV and HIV-1 subtypes: A and D in Uganda. The study also investigated the potential usage of rilpivirine, doravirine and etravirine in patients who failed treatment on efavirenz.

**Methods:**

A retrospective study was conducted on 182 archived plasma samples obtained from patients who were experiencing virological failure between 2006 and 2017 at five Joint Clinical Research Center (JCRC) sites in Uganda. Sanger sequencing of the Reverse Transcriptase (RT) gene from codons 1–300 was done. Mutation scores were generated using the Stanford University HIV Drug Resistance Database. A Chi-square test was used to determine the association between drug resistance mutations (DRMs) and drug regimens or HIV-1 subtypes.

**Results:**

The prevalence of DRMs was 84.6% among patients failing a first-line efavirenz (EFV)-based regimen. The most prevalent Nucleoside Reverse Transcriptase Inhibitor (NRTI) mutations were M184V/I (67.3%), K219/Q/E (22.6%) and K65R (21.1%). While K103N (50.8%) and G190A/S/E/G (29.1%) were the most prevalent Non-Nucleoside Reverse Transcriptase Inhibitor (NNTRI) mutations. As expected, discriminatory DRMs such as K65R, L74I, and Y115F were noted in Tenofovir (TDF) containing regimens while the Thymidine Analogue Mutations (TAMs) L210W and T215 mutations were in Zidovudine (AZT)-based regimens. No significant difference (p = 0.336) was found for overall DRMs between HIV-1 subtypes A and D. Among the patients who had resistance to EFV, 37 (23.6%) were susceptible to newer NNRTIs such as Rilpivirine and Etravirine.

**Conclusion:**

Accumulation of DRMs between AZT/3TC/EFV and TDF/3TC/EFV is comparable but individual mutations that confer resistance to particular drugs should be considered at virological failure. Having either HIV-1 subtype A or D is not associated with the acquisition of DRMs, therefore HIV diversity should not determine the choice of treatment. Rilpivirine, etravirine and doravirine had minimal benefits for patients who failed on efavirenz.

## Background

Globally, a total of 38.9 million (31.1–43.9 million) people are living with HIV while in Sub-Saharan Africa, a total of 19.6 million (17.5–22.0 million) are living with the virus [1]. In Uganda currently, there are 1.6 million living with HIV [2]. It is undeniable that anti-retroviral drugs have played a tremendous role in controlling the epidemic resulting in a significant reduction in AIDS-related deaths and individuals living more prolonged and productive lives. Three or four anti-retroviral drugs are combined into a multi-drug regimen called highly active antiretroviral therapy (HAART) [3] which can suppress HIV to levels below the limits of detection [4, 5]. However, even with the documented success of HAART there are still challenges especially in the low-income countries where there is not only an extreme limitation to the available drug regimens but also adherence to these regimens as well as close monitoring of response to treatment are still lacking [6, 7].

ART regimens have been reported to differ in their abilities to successfully achieve viral suppression [8]. Furthermore, individual drugs within a regimen display differences in genetic barrier to resistance [9] hence regimens that require fewer key mutations to render treatment ineffective have a low genetic barrier to resistance. Such drugs are associated with increased virological failure and development of resistance. Examples of low genetic barrier regimen include non-thymidine combination regimens (e.g. abacavir/lamivudine/tenofovir (ABC/3TC/TDF) and didanosine (ddI/3TC/TDF) [10–12]. On the other hand, regimens with a high genetic barrier to resistance [e.g. boosted protease inhibitors (PIs) give sustained viral suppression and resistance to such drugs develops over a prolonged period. However, these drugs may be compromised by other factors such as adverse drug events or other treatment-limiting factors (e.g. lipid alterations)] [9]. Notably, some of the regimens with a low genetic barrier are used in Uganda’s treatment guidelines, these contain nucleoside backbones (such as TDF/3TC or AZT/3TC) in combination with EFV, boosted PIs or Integrase inhibitors [11]. Despite their use, at virologic failure, it appears that TDF/3TC-containing regimens fail with M184V plus K65R [9] whereas AZT-containing regimens fail with the occurrence of Thymidine Analog Mutations (TAMs) [13, 14]. These differences in drug resistance mutation profiles account for the varying virological outcomes [8, 9, 11, 15, 16]. However, there is a paucity of information regarding treatment outcomes for the currently used treatment regimens. It is well known that the success of any ART regimen depends on how well it attains and sustains viral suppression, therefore, a clear understanding of the drug resistance mutation profiles among first-line drug regimens will give guidance on the best choice of regimen to clinicians and policymakers.

Most of the studies on which treatment guidelines are based are mainly HIV-1 subtype B. However, this subtype accounts for only 12% of worldwide infections and is almost nonexistent in many low to middle-income countries where close 90% of the infection is found [17]. In Uganda, HIV-1 subtypes A and D are the most prevalent [18–20]. There is a growing body of evidence indicating subtype differences about antiretroviral resistance. It has been shown that resistance to ART occurs more in HIV-1 subtype D than in subtype A [21]. Furthermore, resistance to NRTIs, NNRTIs, and PIs vary among the different subtypes [22–26] for example the K65R mutation develops much faster in subtype C than other subtypes [27].

In the low to middle-income countries (LMICs), Efavirenz is still an alternative NNRTI for first-line regimens especially when there is a restriction to dolutegravir (DTG) prescription. However, at virological failure, efavirenz develops mutations that cause cross-resistance to almost the entire NNRTI class [28–30] making it impossible to choose a second line NNRTI containing regimen. Newer NNRTIs such as rilpivirine, doravirine and etravirine are cheaper than protease inhibitors and integrase inhibitors [32] and were designed to circumvent the resistance mechanisms through “conformational flexibility” hence they alter their shape and position to bind to the binding pocket that already contains NNRTI resistance mutations [33–36]. Therefore, rilpivirine, etravirine and doravirine are probable options as second-line ARV agents in LMICs.

In this study, we set out to compare the response to therapy between two commonly used drug regimens: AZT/3TC/EFV and TDF/3TC/EFV and to further analyze EFV based failures to see if there is a possibility of using one of the newer NNRTIs as an alternative.

## Methods

### Study selection criteria

A retrospective study was conducted on 182 archived plasma samples from patients who had a virological failure [HIV viral load above 1000 copies/ml after at least one (1) year on treatment] while taking either TDF/3TC/EFV or AZT/3TC/EFV at the Joint Clinical Research Centre, Kampala, Uganda. All samples that met the inclusion criteria of no prior exposure to ART and a viral load above 1000 copies/ml over 10 years (between 2006 and 2017) were included in the analysis. These samples were collected for routine (once in a year) monitoring of patients’ response to treatment after ART initiation. Patients’ duration on ART, immunological and adherence data were not available. Laboratory assigned sample IDs were used as unique identifiers to maintain confidentiality. The study was carried out under IRB approval (protocol, EM-10-07) at Center for AIDS Research laboratory, Joint Clinical Research Center, Kampala Uganda.

### Laboratory procedures

Viral RNA was extracted from plasma using the Qiagen QIAamp Viral RNA Mini Extraction Kit (Qiagen Inc, Germantown MD) according to the manufacturer’s instructions [38]. Reverse transcriptase-PCR was performed using single-step Superscript III with platinum Tag high DNA polymerase to amplify a 750 base pair fragment of the reverse transcriptase region on the HIV genome [37]. Visualization of PCR product was done using Invitrogen™ SYBR™ Safe DNA Gel Stain [39]. PCR products were purified using ExoSAP-IT™ reagent [40]. The cleaned PCR products were then sequenced using BigDye Terminator v3.1 cycle sequencing kit and sequenced codons 1–248 of the Reverse transcriptase gene using the ABI genetic analyzer 3730xl [41].

### Data analysis

Sequences generated were edited using an online DNA editing software Recall [42], the edited sequences were imported into the Stanford HIV Drug resistance Database Version 8.9 (https://hivdb.stanford.edu/) [43] to generate drug resistance profiles and HIV-1 subtypes. The subtypes were further proofread using COMET software [44]. Statistical analysis was done using IBM SPPS statistics version 25 [45], in which a Chi square test set at significance level (p < 0.005) was used to determine the association between drug resistance mutations and two main factors; Drug regimens (TDF/3TC/EFV and AZT/3TC/EFV) and HIV-1 subtypes (A and D).

## Results

A total of 182 samples were successfully analyzed. The average age of patients was 29.1 ± 13.4 years. Among the participants, 89 (48.9%) were female and 78 (42.9%) were male, the rest of the patients had sex data missing. The patients were in two groups based on the treatment regimen, 101 (55.5%) were on TDF/3TC/EFV and 81 (44.5%) were on AZT/3TC/EFV. The average viral load was 203,072.29 (1287-3910237) copies/ml, Table 1.

**Table 1:** Baseline characteristics of patients

Patient characteristic	
	Average
Age	29.074 ± 13.4349
Viral load	203,072.29 ± 495,549.874

	*n (%)*
Drug regimen	
AZT/3TC/EFV	81 (44.5)
TDF/3TC/EFV	101 (55.5)
Subtypes	
A	90 (49.5)
D	64 (35.2)
Others	28 (15.4)
Sex	
Male	78 (42.9)
Female	89 (48.9)

### Prevalence of drug resistance mutations

**Fig. 1:**
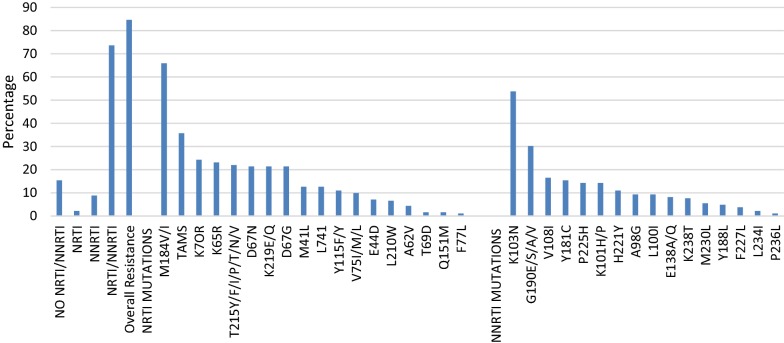
Prevalence of drug resistance mutations

The study had two classes of drugs: the NRTI and the NNRTI class. Overall resistance to at least one class of drugs occurred in 154 (84.6%) of the participants. Resistance to NNRTI class only occurred in 16 (8.8%), NRTI class only occurred in 4 (2.2%) and resistance to both classes occurred in 134 (73.6%) of the participants. When we compared individual mutations within the NRTI class, M184V/I mutation had the highest prevalence of 120 (65.9%) among patients. This was followed by K70R, K65R and K219 mutations occurring at 56 (24.3%), 42 (23.1%) and 40 (22%) respectively. On the other hand, the least frequent NRTI mutations were F77L that occurred in 2 (1.1%) of the patients followed by Q151M and T69D at 3 (1.6%). Comparisons of individual mutations within the NNRTI class showed the most frequent mutation to be K103N; this occurred in 98 (53.8%) of the patients. This was followed by G190A/S/E/G that occurred in 55 (30.2%) of the patients, P236L mutation occurred in 2 (1.1%) and L234I that occurred in only 4 (2.2%) of the participants (Fig. 1).

### Drug resistance mutation profiles between drug regimen

**Fig. 2:**
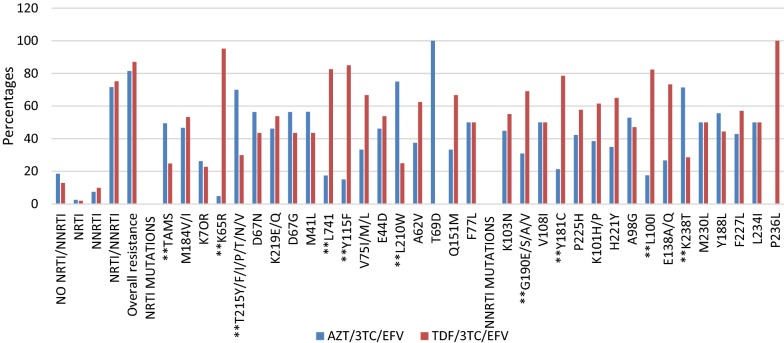
Drug resistance mutation profiles between drug regimen. **Significant difference across drug regimens

A comparison of overall drug resistance profiles between the two drug regimens was not statistically significant. However, comparisons for individual mutations within the NRTI class of drugs indicated that mutations K65R, L74I, Y115F, L210W and T215 mutations differed significantly among the two regimens. K65R (95.2%, p = 0.00005), Y115F (85%, p = 0.005) and L74I (82.6%, p= 0.005) mutations appeared more in the TDF/3TC/EFV group as compared to AZT/3TC/EFV group. As expected, L210W (75%, p = 0.03) and T215 mutations (70%, p = 0.0005) were significantly noted more in AZT/3TC/EFV as compared to TDF/3TC/EFV group. Within the NNRTI class, G190 mutations (69.1%, p = 0.015), Y181C (78.6%, p = 0.008), L100I (82.4%, p = 0.019) were significantly more in the TDF/3TC/EFV group whereas mutation K238T (71.4%, p = 0.035) was significantly more in the AZT/3TC/EFV group (Fig. 2).

### Drug resistance mutation profiles between HIV-1 subtypes A and D

**Fig. 3:**
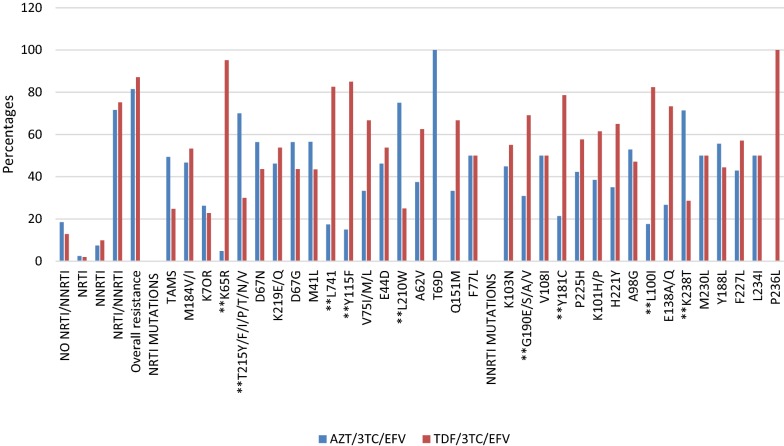
Drug resistance mutation profiles among subtypes. **Significant difference across HIV-1 subtypes

On comparing the overall resistance profiles between HIV-1 subtypes, there were no significant associations between the most predominant HIV-1 subtypes found in Uganda. However, when we compared individual mutations we found that M184V/I (p = 0.015), Y188L (p = 0.008) and TAMs (p = 0.011) were significantly more common in Subtype A as compared to Subtype D and others (Fig. 3).

### Predicted drug susceptibility scores of efavirenz, doravirine, rilpivirine and etravirine

**Fig. 4:**
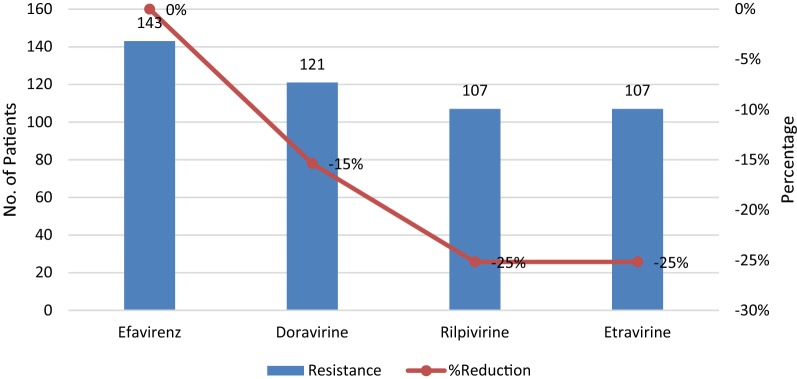
Percentage reduction of efavirenz resistant for rilpivirine doravirine and etravirine

NNRTI drugs are often compromised by cross-resistance within the class, but with the introduction of newer NNRTIs such as rilpivirine, doravirine and etravirine which offer protection even in the presence of resistant variants, there may still be hope for use of some of these drugs in this class. In this study, we compared the predicted drug susceptibility scores of efavirenz together with those of newer NNRTIs. Results showed that out of the 182 patients, 143 (78.5%) had high-level resistance to efavirenz. These patients who had high-level resistance to efavirenz were selected for assessment of drug susceptibility to rilpivirine, doravirine and etravirine to compare percentage reduction in resistance. Of these 36 (25.2%) were susceptible to etravirine and rilpivirine, 22 (15.4%) were susceptible to doravirine, Table 2 resulting into a 15% reduction in resistance for doravirine and a 25% reduction in resistance for etravirine and rilpivirine regardless of the level (Fig. 4).

**Table 2:** Predicted drug susceptibility scores of efavirenz, doravirine, rilpivirine and etravirine

Efavirenz	Susceptible	Low-level resistance	Intermediate resistance	High-level resistance
	Etravirine			
Susceptible (36)	34 (94.4%)	2 (5.6%)	0 (0%)	0 (0%)
Low level resistance (1)	1 (100%)	0 (0%)	0 (0%)	0 (0%)
Intermediate resistance (2)	1 (50%)	0 (0%)	1 (50%)	0 (0%)
High level resistance (142)	36 (25.2%)	33 (23.2%)	49 (34.5%)	25 (17.6%)
Total (182)	72 (39.2%)	35 (19.3%)	50 (27.6%)	25 (13.8%)
	Rilpivirine			
Susceptible (36)	34 (94.4%)	2 (5.6%)	0 (0%)	0 (0%)
Low level resistance (1)	1 (100%)	0 (0%)	0 (0%)	0 (0%)
Intermediate resistance (2)	1 (50%)	0 (0%)	1 (50%)	0 (0%)
High level Resistance (142)	36 (25.2%)	33 (23.2%)	49 (34.5%)	25 (17.6%)
Total (182)	72 (39.2%)	35 (19.3%)	50 (27.6%)	25 (13.8%)
	Doravirine			
Susceptible (36)	36 (100%)	0 (0%)	0 (0%)	0 (0%)
Low level resistance (1)	1 (100%)	0 (0%)	0 (0%)	0 (0%)
Intermediate resistance (2)	1 (50%)	0 (0%)	1 (50%)	0 (0%)
High level Resistance (142)	22 (15.4%)	23 (16.1%)	54 (37.8%)	44 (30.8%)
Total (182)	60 (33%)	23 (12.6%)	55 (30.2%)	44 (24.2%)

## Discussion

Findings from this study have shown that 84.6% of the patients had drug resistance mutations (DRMs) conferring resistance to at least one class of drugs and 73.6% had resistance to both NRTI and NNRTI classes of drugs. Similar studies in sub-Saharan countries had a prevalence of DRMs at 70% [46] and in South Africa, the prevalence of DRMs was at 86% [47]. Recent reports from studies done in Uganda show prevalence at 95.2% [48] and sub-Saharan countries at 98% [26]. The study further noted that within the NRTI class, M184V/I mutations had the highest prevalence of 65.9% similar to a study done in Uganda [48] where M184V was noted with the highest prevalence of 81.2%. The high prevalence of M184V might be attributed to the continued usage of lamivudine as a backbone in all regimens in Uganda and all over sub-Saharan since the presence of this mutation reduces the viral replicative fitness and increases susceptibility to TDF and AZT [49, 50]. Furthermore, Lamivudine has a low genetic barrier.

TAMs and K65R had a prevalence of 35.7% and 23.1% respectively, similar to a study done in Uganda [48] where K65R mutation had a prevalence of 25%. K65R is highly selected for in TDF containing regimens [51]. The high prevalence of K65R might be attributed to the fact that many people had been on a TDF containing regimen for prolonged times and they were beginning to fail. It is also possible that most of these patients do not keep their appointments for scheduled viral load and drug resistance monitoring such that by the time they report back to the clinic, they have been on a failing regimen for quite some time. For NNRTI class, K103N mutation (53.8%) was indicated as the most prevalent mutation followed by G190A/S/E/G (30.2%) similar to a study done in sub-Saharan Africa [48] that indicated K103N (38.7%) and G190A/S/E/G (21.8%) as the most prevalent. This was anticipated given the fact that Efavirenz is highly used as the preferred NNRTI in this region and mutations K103N, G190A are among the most common single mutations that confer high-level resistance to all the first-generation NNRTIs [49].

The current study showed that there is no significant difference (p < 0.367) between AZT/3TC/EFV and TDF/3TC/EFV regimens in attaining DRMs for NRTIs, NNRTIs. The choice of regimen should therefore, be based on other factors other than resistance mutation profiles. These findings are in line with the consolidated guidelines for prevention and treatment of HIV in Uganda which recommends both TDF/3TC and AZT/3TC containing regimens as first-line but TDF/3TC-containing regimens being the preferred first-line [31]. Within the NRTI class, individual mutations differed between these two regimens where mutations K65R (95.2%, p = 0.00005), Y115F (85.0%, p = 0.004) and L74V/I (82.6%, p = 0.004) appeared more frequently in the TDF/3TC/EFV. This is anticipated given the fact that mutation K65R is highly selected for in TDF containing regimens and mutation L74V appears to be associated with poor treatment outcomes in TDF-based regimens [47]. On the other hand, mutation L210W (75%, p = 0.03) and T215 mutations (70%, p = 0.0005) designated as TAMs were significantly more in the AZT/3TC/EFV. This is expected because these are excision mutations [52, 53] and studies have shown that AZT is the most efficiently removed NRTI among all NRTIs by the mutated viral RT enzyme [50]. These mutations confer high-level resistance to AZT [13, 14]. Within the NNRTI class, G190 mutations (38 (69.1%), p = 0.015), Y181C/I/Y (22 (78.6%), p = 0.008) and L1001 (14 (82.4%), p = 0.019) mutations were significantly noted more in TDF/3TC/EFV whereas K238T (10 (71.4%), p = 0.034) noted more in AZT/3TC/EFV regimen. These differences in selection of NNRTI mutation by drug regimen rate TDF/3TC/EFV as an inferior regimen to AZT/3TC/EFV, but these findings can be explained in part by inherent limitations to our retrospective approach, including residual confounding.

Overall, drug resistance mutation profiles had no significant association (p = 0.336) with HIV-1 subtypes as previously reported by Hamers et al. and Venner et al. [46, 54]. The current study in Uganda therefore, suggests that accumulation of resistance mutations is most likely to be due to treatment regimens and other factors e.g. patient adherence. However, M184V/I (67 (55.8%), p = 0.015), Y188L (9 (100%), p = 0.008) and TAMs (44 (51.8%), p = 0.011) were noted more in subtype A, but the number of occurrence of these mutations could not affect the overall association, therefore the study finds these individual mutation observations statistically irrelevant.

The study explored the reliability of newer NNRTIs, rilpivirine, etravirine and doravirine as possible alternatives for future use in patients with high-level resistance to efavirenz. The results revealed that of the 143 patients who had high-level resistance to efavirenz, 107 (74.8%) patients retained high-level resistance to rilpivirine or etravirine and 121 (84.6%) retained resistance to doravirine. The study revealed that only 35 (25.2%) of patients would benefit from rilpivirine or etravirine and 22 (15.4%) from doravirine as options in salvage regimens. These results are in range with findings from a study done in Thailand [55] which showed that 32% of patients who had high-level resistance to efavirenz were susceptible to rilpivirine or etravirine. Similarly, a sub-Saharan study [26] detected resistance to these newer drugs in participants failing on efavirenz (40% etravirine, 51% rilpivirine) and concluded in agreement with the current study that these are unlikely to be considered as options in second-line regimens within the national treatment guidelines.

## Conclusions

This study found no association between AZT/3TC/EFV and TDF/3TC/EFV in attaining resistance mutations NRTIs, NNRTIs suggesting that the choice of regimen should be based on other factors other than resistance mutation profiles. However, there were associations for individual mutations in the TDF-containing regimen and AZT-containing regimen; these, therefore must be put into consideration when choosing a regimen for patients either at baseline or at virological failure. In addition, the study showed that the newer NNRTI; rilpivirine or etravirine or doravirine benefit only a small percentage of people with high-level resistance to efavirenz, therefore are less likely to be considered as options in the national guidelines at second-line for patients who had efavirenz in the first-line regimen. For use in salvage regimen, drug resistance profiles should guide the choice of drugs.

In this study, probable confounders were noted such as patients’ adherence to treatment, time spent on treatment, time to report back for viral load and drug resistance monitoring and the sample size which might have affected the statistical power of the study given the level of occurrence for the individual mutations.

## Data Availability

All data generated or analyzed during this study are included in this published article. Sequences have been deposited within the GenBank under accession numbers MK412157-MK412325.

## Abbreviations

µl: Microliter
AIDS: Acquired Immune Deficiency Syndrome
ARV: Anti-retroviral drugs
CD4+: Cluster of differentiation
CFAR: Center for AIDS Research
HAART: High active antiretroviral therapy
HIV: Human immunodeficiency virus
JCRC: Joint Clinic Research Center
NNRTI: Non-Nucleoside Reverse Transcriptase Inhibitor
NtRTI: Nucleotide Reverse Transcription Inhibitor
NRTI: Nucleoside Reverse Transcriptase Inhibitor
RNA: Ribonucleic acid
RT-PCR: Reverse transcription-polymerase chain reaction
